# Breastfeeding Initiation: Impact of Obesity in a Large Canadian Perinatal Cohort Study

**DOI:** 10.1371/journal.pone.0117512

**Published:** 2015-02-06

**Authors:** Julie Verret-Chalifour, Yves Giguère, Jean-Claude Forest, Jordie Croteau, Peiyin Zhang, Isabelle Marc

**Affiliations:** 1 Departments of Pediatrics, Centre hospitalier universitaire (CHU) de Quebec, Quebec city, Quebec, Canada; 2 Departments of Medical Biology, Centre hospitalier universitaire (CHU) de Quebec, Quebec city, Quebec, Canada; 3 Laboratory of Biostatistics, Centre de recherche de l’institut universitaire en santé mentale de Quebec (Université Laval), Quebec city, Quebec, Canada; TNO, NETHERLANDS

## Abstract

**Objective:**

To evaluate incidence of breastfeeding initiation according to maternal pre-pregnancy body mass index (BMI) in “Grossesse en Santé”, a large prospective birth cohort in Quebec City.

**Methods:**

Breastfeeding initiation in the post-partum period, pre-pregnancy BMI, sociodemographic determinants and obstetrical and neonatal factors were collected from years 2005 to 2010 in 6592 women with single pregnancies. Prenatal non-intention to breastfeed was documented in a subgroup of the cohort (years 2009–2010). Log-binomial regression analyses were performed to assess relative risk (RR) of non-initiation of breastfeeding between maternal BMI categories in models including pre- and post-natal determinants, after exclusion of variables with a mediating effect.

**Results:**

Twenty percent (20%) of obese women did not initiate breastfeeding in the post-natal period at hospital compared to 12% for normal weight women. Compared with those having a normal pre-pregnancy BMI, obese women had a higher risk of non-initiation of breastfeeding (RRunadj 1.69, 95% CI 1.44–1.98), even after adjustment for prenatal and sociodemographic factors (RRadj 1.26, 95% CI 1.08–1.46). Furthermore, the risk of non-initiation of breastfeeding in obese women still remained higher after introduction of per- and post-natal factors (RR 1.22, 95% CI 1.04–1.42). The prenatal non-intention to breastfeed was strongly associated with the non-initiation of breastfeeding for all categories of BMI.

**Conclusion:**

Maternal obesity is associated with a two-fold rate of non-initiation of breastfeeding. Considering the benefits of breastfeeding and the increasing obesity rate, adapted interventions and specialized support should target both pre- and immediate post-natal periods in this population.

## Introduction

The high prevalence of obese women starting pregnancy in Canada (11% to 21%)[1] is a critical challenge for healthcare, considering the tremendous resource and cost implications related to maternal and child morbidity associated with obesity.[2–5] A large body of evidence suggests the influence of obesity morbidities on fetal and perinatal growth and health trajectories.[1, 4, 6, 7][6] Therefore, infants born from mothers with high BMI are more at risk of presenting metabolic disturbances [8] predisposing to a higher risk for metabolic complications later in childhood as diabetes, childhood obesity and hypertension.[9, 10] Despite the potential benefits from breastfeeding for improving these outcomes, [11] obesity is negatively associated with breastfeeding.[12] Obese women are less inclined to breastfeed, [13, 14] initiate it less [12, 15] and the duration of breastfeeding is shorter.[16, 17] Reasons for non-initiation of breastfeeding in obese women are numerous and could be influenced by several factors.

Despite the 2003 World Health Organization and UNICEF infant feeding recommendations [18] endorsed by Canadian Paediatric Society and Health Canada, [19] little information is available on the current situation of breastfeeding in the Canadian obese population. Indeed only one study in Canada [20] reported the impact of maternal obesity on breastfeeding duration in the Canadian population.

The hypothesis of this study was that obese women initiate breastfeeding less often, taking into account their pre- and perinatal characteristics. Using the data of a large prospective populational cohort study (7866 women) recruited in the Quebec City area from 2005 to 2010, the main objective was to determine the incidence of breastfeeding initiation before hospital discharge, according to pre-pregnancy maternal body mass index (BMI). Furthermore, in a sub-group of this cohort (recruited in 2009–2010, 1383 women), we examined the impact of the mother’s prenatal intention of breastfeeding (or not) on the non-initiation of breastfeeding according to the pre-pregnancy maternal BMI.

## Methods

### Setting and study population

We conducted an analysis of breastfeeding initiation by maternal BMI in a birth cohort of 7866 women aged 18 years and more and who delivered in the Quebec City area between March 2005 and April 2010. We restricted our analysis to the 7665 women with no history of total mastectomy and with a singleton live birth who delivered at a gestational age of more than 23 weeks in the two obstetrical centres of level 2 and level 3 in the CHU de Québec. Among these eligible women, those for whom data on pre-pregnancy weight or height were not available (n = 660) were excluded because pre-pregnancy BMI could not be assessed. We also excluded the women for whom information on the primary outcome (i.e. breastfeeding initiation) was missing (n = 413). Thus, analyses were performed in 6592 (86%) of women.

Ethics Statement

The study was approved by the research ethics board of the CHU de Quebec. All subjects gave written informed consent and authorized access to the file for future data collection.

### Design

As described elsewhere, [21] women were recruited at their first prenatal visit at a mean of 14 weeks of gestation. Between 24 and 28 weeks of gestation, they completed a self-administered questionnaire to collect information on socio-demographic factors and medical history. After delivery, mother’s and infant’s charts were systematically reviewed to collect data on pregnancy, delivery and the neonatal period. Diagnostics of pregnancy pathologies, such as preeclampsia or gestational diabetes, were made by specialized physicians in agreement with the recognized diagnostic criteria. [22, 23]

Primary outcome

The primary outcome was breastfeeding initiation, which was systematically collected throughout the in-hospital post-partum period. Breastfeeding initiation (yes/no) was defined as any provision of the mother’s own breast milk (expressed or directly from the breast) to the infant at least once between birth and hospital discharge. [12, 24]

Exposure and other variables

To calculate pre-pregnancy BMI (BMI = weight (kg) / [height (m)]^2^), maternal pre-pregnancy weight and height were collected from the medical records and questionnaires. As we had no ability to determine if anthropometric parameters were more accurate in the questionnaires than in the charts, a mean was calculated to reduce statistical errors (when information was available from only one record, the other one was imputed by regression before calculation of the mean). Pre-pregnancy BMI was grouped into 4 categories [25] underweight (BMI < 18.5 kg/m^2^) normal weight (BMI between 18.5 and 24.99 kg/m^2^), overweight (BMI between 25 and 29.99 kg/m^2^) and obese (BMI ≥30 kg/m^2^).

Covariates were selected for their potential influence in the relation between obesity and breastfeeding, including maternal age, education, marital status, parity, ethnic group, household income, smoking status, alcohol consumption, history of drug use and breastfeeding experience following previous pregnancies. The pairing of ethnicity of the pregnant woman’s parents was used to determine the ethnicity of the pregnant woman. References to mastitis, abscess, breast lump, adenoma, Reynaud’s phenomenon, hyperprolactinemia, and cyst were considered as a minor breast history. Breast reduction or augmentation, breast deformation and little or no gland development in a breast were considered as a major breast history. All these variables were classified as antenatal characteristics. Pre-pregnancy chronic conditions (hypertension, diabetes or other health issues) were not retained in analyses because of their low potential as confounding factors.

Gestational diabetes, hypertensive disorders of pregnancy including high blood pressure, pre-eclampsia and HELLP (Haemolysis, Elevated Liver enzymes and Low Platelet count) syndrome, mode of delivery, anesthesia during labor and gestational age were classified as pernatal information. Finally, neonatal information includes birth weight and percentile, gestational age at birth, apgar at 5 minutes, sex and admission to neonatal unit.

In addition, the mother’s breastfeeding intention before delivery was collected in a subgroup of women who delivered between 2009 and 2010 through a delivery admission questionnaire, including the statement “breastfeeding expected? (yes/no)”. According to the answer, women were classified as “prenatal intention to breastfeed” (yes/no). Breastfeeding intention was missing for 109 women (n = 103 unknown and 6 undecided). Among the 1512 women of the cohort expected to give birth in the 2009–2010 years, the information on BMI, breastfeeding initiation and breastfeeding intention was available for 1174.

### Statistical analyses

The primary goal of this study was to estimate the relative risk (RR) of non-initiation of breastfeeding between obese and normal weight women, through the use of log-binomial regression modelling with adjustment for antenatal characteristics, pernatal and newborn’s characteristics. Another aim was to estimate the RR of non-initiation of breastfeeding for women who had no intention to breastfeed compared to women having the intention to do so. We also wanted to evaluate whether this association may differ between BMI categories by obtaining category-specific non-intention RR and comparing the RR of each category to the RR of the reference category. This was achieved through log-binomial regression, with a model including the non-intention variable, BMI category and the interaction between these two variables, plus the antenatal characteristics. All analyses (including non adjusted RR) were adjusted for the year and place of birth. All analyses were performed using SAS, version 9.3; SAS Institute, Inc.

Continuous covariates of the study had no missing values. However, missing values ranged from 0% to 23% for categorical covariates (mean of 5%), resulting in 49% of the 6592 women eligible for analysis with all covariates available. Restricting our analyses to this subset of women with all data observed would have resulted in a significant loss of information and possibly biased estimations. Therefore, for those women with missing covariates, we used a multiple imputation inference method implemented in SAS and involving three distinct phases. [26, 27]

First, we used the Markov chain Monte Carlo imputation method with single chain implemented in the procedure MI to generate 10 complete data sets. Then we estimated the log-binomial model on the 10 complete data sets separately using the GENMOD procedure. Convergence problems with the natural logarithm link function forced us to replace the binomial distribution by the Poisson distribution with robust variance estimation, a technique known to yield unbiased estimations of the beta coefficients and their variance.[28] The procedure MIANALYZE was finally used to combine the coefficient estimates (and estimations of their variances) from the 10 log-binomial analyses, in order to obtain valid statistical inferences about the model coefficients that take within and between analysis variances into account.[27]

The SAS procedures MI and MIANALYZE require the Missing at Random (MAR) assumption for all analyses. While it is impossible to check its validity, this assumption is more permissive than the Missing Completely at Random (MCAR) assumption which is needed to restrict analyses to the subset of subjects with all covariates available. In order to verify whether a deviation from the MAR assumption could have led to biased inferences in our data, we performed sensitivity analyses by defining two extreme cases of missing value patterns: 1) For each categorical variable with missing values, all missing values are replaced by the category (class) having the most *negative* effect in the analysis done with multiply imputed data; 2) same as 1), except that missing values are replaced by the category (class) having the most *positive* effect.

In order to avoid model overadjustment, seven variables were tested as potential mediators in the relationship between BMI and breastfeeding: Mode of delivery (caesarean or vaginal), gestational hypertension or HELLP or pre-eclampsia (yes or no), type of anesthesia (general anesthesia or other), gestational diabetes (yes or no), transfer to neonatal unit (yes or no), apgar at 5 minutes and weight percentile. Apgar and weight percentile were considered continuously, while the other five were dichotomized. Given the nature of the variables in the context of mediation, analyzes were performed with MPLUS version 7.11. These are logistic regressions with probit link and a WLSMV estimate. Confidence intervals were generated using Boostrap (n = 2000). The WRMR (Weighted Root Mean Square Residual) was used as a model fit index. A value less than 1 indicates a good fit. [40][1]

## Results

### Population characteristics

Based on pre-pregnancy BMI, 4105 women among the 6592 (62.3%) were normal weight. A total of 337 (5.1%) were classified as underweight, 1317 (20.0%) as overweight and 833 (12.6%) as obese. Women’s socio-demographic characteristics according to the BMI are reported in Table 1. Obese women were less educated, had a lower household income and reported more major breast history. They were more often multiparous (two children or more) but compared to multiparous normal weight women, they reported significant shorter experience of breastfeeding following previous pregnancies (Table 1). Indeed 25.9% of obese women did not breastfeed their previous children compared to 17.7% of the normal weight women. Globally, gestational age at delivery ranged from 24 4/7 to 42 6/7 weeks with a mean of 39.37 ±1.54 weeks; there were significantly more preterm deliveries in obese women (Table 2).

**Table 1:** Antenatal characteristics according to pre-pregnancy body mass index.

	Women’s pre pregnancy BMI, n (%)*			
Pre pregnancy BMI	Underweight	Normal weight	Overweight	Obese
kg/m^2^, Mean ±SD	17.7 ± 0.8	21.7 ± 1.7	27.1 ± 1.4	35.1 ± 4.4
Characteristics (n = 6592)	337 (5.1)	4105 (62.3)	1317 (20.0)	833 (12.6)
		(reference)		
Age at delivery, yr				
Mean ±SD	28.9 ± 4.2	30.0 ± 4.3	30.1 ± 4.3	30.1 ± 4.3
*p* value ^†^	0.1769		0.1005	<.0001
Year of delivery				
2005	13 (3.9)	158 (3.9)	56 (4.3)	24 (2.9)
2006	92 (27.3)	985 (24.0)	304 (23.1)	178 (21.4)
2007	110 (32.6)	1328 (32.3)	415 (31.5)	253 (30.4)
2008	66 (19.6)	902 (22.0)	287 (21.8)	188 (22.6)
2009	39 (11.6)	471 (11.5)	159 (12.1)	131 (15.7)
2010	17 (5.0)	261 (6.4)	96 (7.3)	59 (7.1)
*p* value	0.6865	―	0.7592	0.0082
Education				
High school not achieved	26 (8.3)	124 (3.2)	59 (4.8)	43 (5.6)
High school achieved	85 (27.0)	793 (20.7)	318 (26.0)	219 (28.6)
College	106 (33.6)	1238 (32.3)	421 (34.4)	274 (35.7)
University	98 (31.1)	1682 (43.8)	425 (34.8)	231 (30.1)
*p* value	<.0001	―	<.0001	<.0001
Ethnic group of pregnant women				
Caucasian (white)	279 (94.6)	3569 (97.0)	1148 (97.2)	721 (96.5)
Other	12 (4.1)	60 (1.6)	15 (1.3)	10 (1.3)
Multiracial	4 (1.3)	51 (1.4)	18 (1.5)	16 (2.1)
*p* value	0.0104	―	0.6449	0.2601
Marital status				
Single, Separated, Divorced	24 (9.0)	252 (8.0)	69 (6.7)	45 (6.8)
Common-law partner, Married	244 (91.0)	2890 (92.0)	955 (93.3)	622 (93.2)
*p* value	0.5901	―	0.1815	0.2652
Household income, per yr				
< $15 499	24 (8.6)	114 (3.2)	41 (3.6)	30 (4.2)
$15 500 to $24 999	29 (10.4)	210 (5.9)	58 (5.1)	44 (6.2)
$25 000 to $39 999	40 (14.4)	400 (11.2)	162 (14.1)	136 (19.1)
$40 000 to $59 999	56 (20.2)	753 (21.0)	262 (22.8)	183 (25.6)
≥ $60 000	129 (46.4)	2101 (58.7)	624 (54.4)	320 (44.9)
*p* value	<.0001	―	0.0185	<.0001
Parity (Para)				
0	179 (53.1)	2019 (49.2)	609 (46.2)	334 (40.1)
1	124 (36.8)	1595 (38.9)	531 (40.3)	349 (41.9)
≥ 2	34 (10.1)	491 (11.9)	177 (13.4)	150 (18.0)
*p* value	0.3266	―	0.1290	<.0001
Smoking status ^a^				
Ex-smoker	73 (23.0)	1027 (26.6)	351 (28.6)	224 (29.1)
Smoker	59 (18.6)	362 (9.4)	105 (8.5)	95 (12.3)
Non-smoker	185 (58.4)	2469 (64.0)	772 (62.9)	451 (58.6)
*p* value	<.0001	―	0.3304	0.0063
Alcohol consumption, drink per wk ^b^				
0	277 (93.6)	3390 (92.9)	1103 (94.0)	688 (95.3)
≥1	19 (6.4)	260 (7.1)	70 (6.0)	34 (4.7)
*p* value	0.6493	―	0.1726	0.0180
Any history of drug use ^b^				
No	185 (66.8)	2275 (69.7)	747 (70.5)	523 (75.9)
Yes	92 (33.2)	988 (30.3)	313 (29.5)	166 (24.1)
*p* value	0.3086	―	0.6435	0.0012
Major breast history ^c^				
No	334 (99.1)	4034 (98.3)	1275 (96.8)	798 (95.8)
Yes	3 (0.9)	71 (1.7)	42 (3.2)	35 (4.2)
*p* value	0.2471	―	0.0013	<.0001
Minor breast history ^c^				
No	332 (98.5)	4055 (98.8)	1304 (99.0)	827 (99.3)
Yes	5 (1.5)	50 (1.2)	13 (1.0)	6 (0.7)
*p* value	0.6069	―	0.4962	0.2161
Previous breastfeeding experience, mo ^d^				
0	26 (20.5)	266 (17.7)	93 (18.0)	100 (25.9)
1–3	34 (26.8)	344 (22.9)	134 (25.9)	107 (27.7)
4–12	53 (41.7)	680 (45.2)	220 (42.6)	136 (35.3)
≥ 13	14 (11.0)	215 (14.2)	70 (13.5)	43 (11.1)
*p* value	0.6280	―	0.2026	<.0001

Note: BMI = Body mass index; SD = Standard deviation

* Unless stated otherwise

^†^ *p* value indicates statistical difference between each BMI category and reference category for the related characteristic

^a^ At the beginning of actual pregnancy

^b^ During actual pregnancy

^c^ Refers to a previous history of illness or surgery to breast

^d^ Number of months of breastfeeding experimented by women with one or more previous children. Refer exclusively to multiparous women (n = 3451)

**Table 2:** Pernatal characteristics according to pre-pregnancy body mass index (BMI).

	Women’s pre pregnancy BMI, n (%)*			
	Underweight	Normal weight	Overweight	Obese
Characteristic (n = 6592)	337 (5.1)	4105 (62.3)	1317 (20.0)	833 (12.6)
		(reference)		
Gestational age, wk by category				
<33	2 (0.6)	26 (0.6)	9 (0.7)	7 (0.8)
33–36 6/7	23 (6.8)	176 (4.3)	66 (5.0)	53 (6.4)
≥37	312 (92.6)	3903 (95.1)	1242 (94.3)	773 (92.8)
*p* value ^†^	0.0959	―	0.5293	0.0266
Gestational age, wk				
Mean ±SD	39.1 ± 1.6	39.4 ± 1.5	39.4 ± 1.5	39.2 ± 1.7
*p* value	0.1942	―	0.0924	<.0001
Gestational diabetes				
No	322 (97.0)	3903 (96.5)	1207 (92.9)	698 (84.1)
Yes	10 (3.0)	141 (3.5)	93(7.1)	132 (15.9)
*p* value	0.6488	―	<.0001	<.0001
Gestational hypertension or HELLP^a^ or pre-eclampsia				
No	290 (93.6)	3494 (92.8)	1066 (87.2)	594 (77.0)
Yes	20 (6.4)	271 (7.2)	156 (12.8)	177 (23.0)
*p* value	0.6238	―	<.0001	<.0001
Mode of delivery				
Elective caesarean section	22 (6.5)	370 (9.0)	141(10.8)	124 (15.0)
Urgent caesarean section	19 (5.7)	421 (10.3)	160 (12.2)	136 (16.4)
Vaginal	295 (87.8)	3302 (80.7)	1008 (77.0)	569 (68.6)
*p* value	0.0045	―	0.0159	<.0001
Anaesthesia during delivery				
None	54 (18.6)	496 (14.1)	131 (11.4)	61 (8.4)
Other	2 (0.7)	38 (1.1)	6 (0.5)	3 (0.4)
General	2 (0.7)	29 (0.8)	5 (0.4)	13 (1.8)
Epidural	205 (70.7)	2467 (70.0)	846 (73.5)	498 (68.3)
Spinal	27 (9.3)	495 (14.0)	164 (14.2)	154 (21.1)
*p* value	0.0692	―	0.0280	<.0001
Place of birth				
Center 1 (level 3)	89 (26.4)	1251 (30.5)	420 (31.9)	263 (31.6)
Center 2 (level 2)	248 (73.6)	2853 (69.5)	897 (68.1)	569 (68.4)
*p* value	0.1174	―	0.3356	0.5199

Note: BMI = Body mass index; SD = Standard deviation

*Unless stated otherwise

^†^ *p* value indicates statistical difference between each BMI category and the reference category for related characteristic

^a^ HELLP syndrome: Haemolysis, Elevated Liver enzymes, Low Platelet count

Obese women also experienced more often gestational diabetes, hypertensive disorders of pregnancy, caesarean section and general anesthesia (Table 2). Infants from obese women had significantly larger birth weight for gestational age and had a higher risk of neonatal morbidities warranting admission to the neonatal intensive care unit (Table 3).

**Table 3:** Newborn’s characteristics according to maternal pre-pregnancy body mass index.

	Women’s pre pregnancy BMI, n (%)*			
	Underweight	Normal weight	Overweight	Obese
Characteristic (n = 6592)	337 (5.1)	4105 (62.3)	1317 (20.0)	833 (12.6)
		(reference)		
Child’s weight percentile				
SGA (<10)	38 (11.3)	238 (5.8)	60 (4.6)	31 (3.7)
APA (10–90)	285 (84.6)	3550 (86.5)	1076 (81.8)	649 (78.1)
LGA (>90)	14 (4.1)	315 (7.7)	179 (13.6)	151 (18.2)
*p* value ^†^	<.0001	―	<.0001	<.0001
Baby’s weight, g				
Mean ±SD	3227 ± 475	3393 ± 470^a^	3502 ± 498^a^	3526 ± 564^a^
*p* value	0.2438	―	0.0668	<.0001
Apgar at 5 minutes				
0–3	0 (0)	4 (0.1)	2 (0.1)	2 (0.2)
4–7	10 (3.0)	70 (1.7)	26 (2.0)	20 (2.4)
8–10	327 (97.0)	4017 (98.2)	1286 (97.9)	805 (97.4)
*p* value	0.2203	―	0.6285	0.1583
Child’s sex				
Boy	168 (49.9)	1933 (47.1)	610 (46.4)	403 (48.6)
Girl	169 (50.1)	2167 (52.9)	705 (53.6)	427 (51.4)
*p* value	0.3390	―	0.6315	0.4588
Transfer to neonatal unit or nursery				
No	314 (96.6)	3806 (95.6)	1215 (94.8)	744 (92.1)
Yes	11 (3.4)	176 (4.4)	67 (5.2)	64 (7.9)
*p* value	0.3785	―	0.2314	<.0001
Breastfeeding initiation				
No	49 (14.5)	494 (12.0)	181(13.7)	167 (20.1)
Yes	288 (85.5)	3611 (88.0)	1136 (86.3)	666 (79.9)
*p* value	0.1770	―	0.1021	<.0001

Note: BMI = Body mass index; SD = Standard deviation; SGA = Small of gestational age; APA = appropriate for gestational age; LGA = Large for gestational age

*Unless stated otherwise

^†^ *p* value indicates statistical difference between each BMI category and the reference category for the related characteristic

^a^ One value is missing

### Breastfeeding outcomes

Overall, 5701 women out of 6592 (86.5%) initiated breastfeeding at least once before hospital discharge. As compared to the normal weight group where only 12% did not initiate breastfeeding, 20% of the women in the obese group did not breastfeed (Table 3).

Therefore, obese women had a higher risk of non-initiation of breastfeeding compared to normal weight women, as evidenced by the unadjusted RR_unadj_ = 1.69 (95% CI 1.44–1.98) (Table 4). Risk for non-initiation of breastfeeding was still significantly higher in obese women after adjustment for antenatal characteristics (RR_ante-adj_ = 1.26, 95% CI 1.08–1.46). In addition, pernatal and newborn’s characteristics also played an important role in the association between BMI and breastfeeding initiation. Among the perinatal potential mediators evaluated through mediation analysis, only mode of delivery and gestational diabetes had significant indirect effects in the association between obesity and breastfeeding non-initiation (both had a positive effect with respective *p* values of 0.014 and 0.003). Type of anesthesia (*p* value = 0.071, 95% CI 0.011–0.173) and transfer to neonatal unit (*p* value = 0.098, 95% CI 0,001–0.056) do not show *p* values less than 0.05 but present significant confidence intervals, allowing to also consider these two variables as mediators.

**Table 4:** Relative risk of non-initiation of breastfeeding associated with maternal pre pregnancy BMI.

Maternal pre-pregnancy BMI	RR (95% CI)	*p* value
RR Unadjusted^a^		
Underweight	1.19 (0.91–1.56)	0.208
Normal weight	Ref	—
Overweight	1.15 (0.98–1.35)	0.085
Obese	1.69 (1.44–1.98)	<.0001
RR Adjusted for place, year of delivery and antenatal^b^ characteristics		
Underweight	0.98 (0.76–1.26)	0.847
Normal weight	Ref	—
Overweight	1.07 (0.92–1.25)	0.381
Obese	1.26 (1.08–1.46)	0.003
RR Adjusted for place, year of delivery, antenatal^b^, pernatal^c^ and newborn^d^ characteristics		
Underweight	0.97 (0.75–1.25)	0.801
Normal weight	Ref	—
Overweight	1.07 (0.91–1.24)	0.423
Obese	1.22 (1.04–1.42)	0.013

Note: BMI = Body mass index; CI = confidence interval; RR = Relative risk; Ref = Reference group

^a^ except for place and year of delivery

^b^ Includes age at delivery, ethnic group, marital status, education, household income, smoking status, major or minor breast history, parity, alcohol consumption during pregnancy, any history of drug use and previous breastfeeding experience

^c^ Includes gestational hypertension or HELLP or pre-eclampsia and gestational age at delivery

^d^ Includes newborn’ sex, weight, weight percentile, and apgar at 5 minutes

We thus excluded these four mediators from the log-binomial analysis model including antenatal, pernatal and newborn’s characteristics as confounding variables. Taking into account all other factors, obesity remained positively associated with non-initiation of breastfeeding (RR_peri-adj_ = 1.22, 95% CI 1.04–1.42). This suggests a significant role of obesity on breastfeeding initiation at birth, even when adjusting for the socio-demographic characteristics and non-mediating obstetrical and neonatal characteristics. There were no such associations when underweight or overweight women were compared to normal weight women.

Sensitivity analyses led to results very similar to the analyses performed on multiply imputed data and none of them led to different conclusions (S1 Table & S2 Table). For example, the two sensitivity analyses for the model including antenatal, prenatal and newborn’s characteristics as confounding variables, both yielded relative risks differing by less than 5% compared to RR_peri-adj_ reported above (1.28, 95% CI 1.09–1.49 and 1.28, 95% CI 1.10–1.50).

Finally, in the subgroup of participants (n = 1174) where prenatal intention to breastfeed was assessed (yes/no), results indicated that the prenatal non-intention to breastfeed was strongly associated with the non-initiation of breastfeeding for all categories of BMI even after adjustment for antenatal women’s characteristics (underweight women RR = 7.242, 95% CI 2.012–26.060; normal weight women RR = 18.202, 95% CI 10.667–31.057; overweight women RR = 8.088, 95% CI 3.836–17.054; obese women RR 10.674, 95% CI 5.140–22.167). The difference in RR was only statistically significant in overweight women compared with normal weight (p = 0.048).

## Discussion

Despite recent recommendations, obese women are less likely to initiate breastfeeding compared to normal weight in the immediate post-partum period. Furthermore identifying obese women as a population at risk, the results from this cohort provide insight into the impact of mother’s socio-demographic determinants, and some perinatal and child’s characteristics on the association between obesity and breastfeeding initiation. This is of major clinical relevance because of the potential protective effect of breastfeeding on child growth and development, and the need to identify targets and timing for interventions in the context of maternal obesity. [9–11]

These results are in accordance with other large observational studies in industrialized countries indicating that obese women were also considered as “at risk” of non-initiation of breastfeeding [12–17] as highlighted in a recent review, [14] including Australian populations (N = 3075, OR 2.10, 95% CI 1.49–2.96 adjusted for socio-demographic factors, caesarean birth and admission to neonatal unit), [16] and American populations (N = 1 161 949, OR 0.84, 95% CI 0.83–0.85 adjusted for socio-demographic factors, parity, gestational age, year and mode of delivery, infant birth weight and sex). [29] One Canadian cohort suggested obesity as a risk factor for a shorter duration of breastfeeding in this population compared with normal weight women (OR 2.39, 95% CI 1.48–3.88, N = 780).[20] While at high risk for perinatal complications, our results strongly demonstrate their role on early breastfeeding initiation in obese women. Our mediation analyses reveal that this association between obesity and breastfeeding non-initiation is partially mediated by mode of delivery, gestational diabetes, type of anesthesia and transfer to neonatal unit. In other words, these factors are in the causal pathway between obesity and higher risk of non-initiation of breastfeeding. Several other factors are potentially involved. Indeed, large breasts and difficulties of movement are two important limitations that complicate breastfeeding initiation [30] and cause women to need extra help.[31] Moreover, delayed lactogenesis may occur in obese women due to influence of fat mass on prolactin and ocytocin.[12] Consequently a woman with a past history of delayed lactogenesis related to a failure to breastfeed may be less motivated to initiate breastfeeding again, a factor that we took into consideration in our analyses. Furthermore our study suggests that a prenatal intention to breastfeed was positively associated to the initiation of breastfeeding whatever the BMI category. It suggests that among women who did not initiate breastfeeding, this non-initiation came also from mother’s choice. Although the differences were not statistically significant for obese women, the strength of this association was lower in obese and overweight compared with normal weight women. It means that there is probably a higher proportion of obese and overweight women for whom it was not their pre-pregnancy choice when they did not initiate breastfeeding. This suggests that the power of prenatal intention may be lower in obese population, but this needs to be clarified in subsequent studies.

The major strength of our study is the large sample size. It is also one of the first to take into account the effects of such a large number of maternal and child relevant factors. Moreover, because francophone Canadian women had a history of lower rate of breastfeeding compared to English Canadian women, [24, 32] it is important to report data in this population following the recent WHO recommendation. However, our data failed to include psychological factors such as body perception, known to be poor in obese women, [33–35] social support [36] and familial perception of breastfeeding, [37] which are identified as factors affecting success of breastfeeding. Social knowledge of, social influence toward, maternal confidence in, and behavioral beliefs about breastfeeding have been recently reported to influence breastfeeding outcomes. [34]

Some readers may view a source of concern in our use of the Missing at Random (MAR) assumption in the statistical analyses, which is required by the SAS procedures MI and MIANALYZE. It is never possible to check the validity of this assumption. However, restraining our analyses to the subset of subjects with all covariates available would require that our data follow a pattern Missing Completely at Random (MCAR)–an assumption more stringent than MAR—in order to obtain unbiased inferences. Moreover, this would entail a great loss of information and thus confidence intervals would widen significantly. Sensitivity analyses gave us more confidence that our conclusions are valid, despite the fact that we don’t know whether the data is MAR.

In accordance with ministerial policies, successful breastfeeding needs to become a cultural norm especially among specific populations such as obese women and their children. [38] Our study provides information by determining that obese women initiate twice less often breastfeeding in the immediate post-partum period than normal weight women. Health professionals can play a major role for this population at risk of non-breastfeeding, by adapting clinical advices and ensuring a more personalized professional help to them. Clinician must be sensitive to the obstetrical and physical experience of obese women linked to initiation of breastfeeding. Further research about exclusive breastfeeding is needed because literature suggests that even when an obese woman initiates breastfeeding, babies often receive supplementation that can lead to a shorter duration of breastfeeding.[12, 39] However, breastfeeding initiation is the beginning of the lactation experience and is a major component to the success of long-term breastfeeding.

## Supporting Information

S1 Table Sensitivity analysis of our log-binomial regressions on the multiply imputed data. Case 1: For each categorical variable with missing values, all missing values are replaced by the category (class) having the most *negative* effect in the analysis done with multiply imputed data.
(DOCX)

S2 Table Sensitivity analysis of our log-binomial regressions on the multiply imputed data. Case 2: For each categorical variable with missing values, all missing values are replaced by the category (class) having the most *positive* effect in the analysis done with multiply imputed data.
(DOCX)
