# Supplementary material for: Breastfeeding Initiation: Impact of Obesity in a Large Canadian Perinatal Cohort Study
Source: PLoS One. 2015 Feb 6;10(2):e0117512. doi: 10.1371/journal.pone.0117512 (PMC4320116; doi:10.1371/journal.pone.0117512)
Supplement: S1 Table — Case 1: For each categorical variable with missing values, all missing values are replaced by the category (class) having the most negative effect in the analysis done with multiply imputed data. (DOCX) [file pone.0117512.s001.docx]

| **Supplementary table 1. Sensitivity analysis of our log-binomial regressions on the multiply imputed data. Case 1:  For each categorical variable with missing values, all missing values are replaced by the category (class) having the most *negative* effect in the analysis done with multiply imputed data** | | |
| --- | --- | --- |
| Maternal pre-pregnancy BMI | RR (95% CI) | *p* value |
| ***RR Unadjusted^a^*** |  |  |
| Underweight | 1.19 (0.91-1.56) | 0.208 |
| Normal weight | Ref | ― |
| Overweight | 1.15 (0.98-1.35) | 0.085 |
| Obese | 1.69 (1.44-1.98) | <.0001 |
| ***RR Adjusted for place, year of delivery and antenatal^b^ characteristics*** | | |
| Underweight | 0.99 (0.77-1.28) | 0.941 |
| Normal weight | Ref | ― |
| Overweight | 1.09 (0.94-1.27) | 0.257 |
| Obese | 1.32 (1.14-1.54) | 0.0002 |
| ***RR Adjusted for place, year of delivery, antenatal^b^, pernatal^c^ and newborn^d^ characteristics*** | | |
| Underweight | 0.98 (0.76-1.26) | 0.852 |
| Normal weight | Ref |  |
| Overweight | 1.09 (0.93-1.27) | 0.276 |
| Obese | 1.28 (1.10-1.50) | 0.0015 |

Note: BMI = Body mass index; CI = confidence interval; RR = Relative risk; Ref = Reference group

^a^ except for place and year of delivery

^b^ Includes age at delivery, ethnic group, marital status, education, household income, smoking status, major or minor breast history, parity, alcohol consumption during pregnancy, any history of drug use and previous breastfeeding experience

^c^ Includes gestational hypertension or HELLP or pre-eclampsia and gestational age at delivery

^d^ Includes newborn’ sex, weight, weight percentile, and apgar at 5 minutes
